# Clinical spectrum, immune status, and prognostic factors of cryptococcosis: insights from a large, multi-center, ambispective cohort study in southeastern China

**DOI:** 10.1186/s40249-025-01408-3

**Published:** 2026-01-04

**Authors:** Lei Gu, Jing Lin, Anmao Li, Jian Yue, Wen Wen, Wei Liu, Qunying Lin, Xiangqi Chen, Xiaohong Chen, Jun Wu, Zeyi Liu, Baosong Xie, Guoxiang Lai, Jian-an Huang

**Affiliations:** 1 Department of Pulmonary and Critical Care Medicine, The First Affiliated Hospital of Soochow University, Suzhou, 215006 China; 2 Department of Infectious Diseases, The First Affiliated Hospital of Soochow University, Suzhou, 215006 China; 3 Department of Rheumatology and Immunology, Affliated Hospital of Zunyi Medical University, Zunyi, 563000 China; 4 The People’s Hospital of Gaozhou, Gaozhou, 525200 China; 5 Department of Pulmonary and Critical Care Medicine, Fuzong Clinical Medical College of Fujian Medical University, Dongfang Hospital of Xiamen University, School of Medicine, Xiamen University, 900th Hospital of PLA Joint Logistic Support Force, Fuzhou, 350001 China; 6 Department of Pulmonary and Critical Care Medicine, The Affiliated Hospital of Putian University, Putian, 351100 China; 7 Department of Pulmonary and Critical Care Medicine, Fujian Medical University Union Hospital, Fuzhou, 350001 China; 8 Department of Pulmonary and Critical Care Medicine, Fuzhou Pulmonary Hospital of Fujian, Fujian Medical University Clinical Teaching Hospital, Fuzhou, 350001 China; 9 Department of Pulmonary and Critical Care Medicine, The Second Hospital of Nanping, Nanping, 353000 China; 10 Department of Pulmonary and Critical Care Medicine, Fuzhou University Affiliated Provincial Hospital, Fuzhou, 350001 China; 11 Department of Pulmonary and Critical Care Medicine, The Second Affiliated Hospital of Fujian University of Traditional Chinese Medicine, Fuzhou, 350003 China

**Keywords:** Cryptococcosis, Immune status, Cryptococcal meningitis, Prognosis, Cryptococcal antigen, Antifungal therapy

## Abstract

**Background:**

Cryptococcosis is a major opportunistic fungal infection with heterogeneous clinical outcomes; however, data on clinical features and prognostic factors in non-HIV populations remain limited. This study aimed to provide real-world evidence on the clinical characteristics, immune stratification, diagnostic performance, treatment patterns, and outcomes of cryptococcosis.

**Methods:**

We performed a multi-center ambispective cohort study of patients with cryptococcosis diagnosed between 2013 and 2025 across 48 hospitals in southeastern China, including Jiangsu and Fujian provinces. Patients were stratified according to immune status, disease type, and prognosis. Categorical variables were compared using the chi-square test or Fisher's exact test, and continuous variables were analyzed using the Mann-Whitney U test or Kruskal-Wallis test, as appropriate.

**Results:**

A total of 396 patients were included, with a median age of 52 years; 61.9% were male. Most patients were immunocompetent (57.1%), while 33.1% had mild and 9.9% severe immunodeficiency. Pulmonary disease predominated (89.7%), whereas 10.1% had meningitis/dissemination. Severe immunodeficiency (SID) was associated with fever, neurological symptoms, lymphopenia, and elevated C-reactive protein (CRP) (all *P* < 0.01). Patients with meningitis/dissemination had more neurological manifestations and a markedly worse prognosis than those with pulmonary disease (mortality 35.1% vs. 2.1%). Among 319 patients with available follow-up data, follow-up duration varied from several days to several years, with prospective patients followed for up to 12 months. Overall, 89.0% recovered or improved, while 6.0% deteriorated or died. Poor outcomes were associated with older age, SID, central nervous system (CNS) involvement, lymphopenia, and elevated CRP. Serum cryptococcal antigen (CrAg) assays showed 94.6% concordance (122/129) between qualitative and quantitative methods. Quantitative ELISA identified four additional positive cases but missed three qualitative positive cases. In pulmonary cryptococcosis, amphotericin B-containing regimens were rarely used, while azole monotherapy was administered to over 95% of patients across severity groups and achieved favorable outcomes.

**Conclusions:**

Host immune status, CNS involvement, and systemic inflammation are key predictors of outcome in cryptococcosis. Quantitative and qualitative CrAg assays demonstrate high diagnostic performance and azole monotherapy remained effective for pulmonary disease. These findings support risk-stratified diagnostic and therapeutic strategies in routine clinical practice, particularly in resource-limited settings.

**Graphical abstract:**

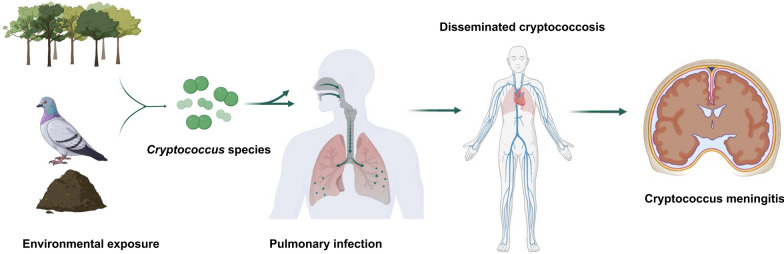

**Supplementary Information:**

The online version contains supplementary material available at 10.1186/s40249-025-01408-3.

## Background

Cryptococcosis is a life-threatening opportunistic fungal infection caused by *Cryptococcus* species belonging to the *C. neoformans* and *C. gattii* complex—now classified into seven species—among which *C. neoformans* and *C. gattii* remain the most clinically significant pathogens. It has a global distribution, with an estimated 152,000 cases of cryptococcal meningitis and 112,000 associated deaths annually, predominantly affecting people living with HIV, while an increasing proportion of cases are now recognized in non-HIV populations [1]. Although classically associated with advanced HIV infection, cryptococcosis is increasingly recognized in non-HIV populations, including transplant recipients, patients receiving immunosuppressive therapy, and individuals with chronic comorbidities [2, 3].

The clinical spectrum of cryptococcosis is highly variable, ranging from asymptomatic pulmonary nodules to severe meningoencephalitis with poor prognosis. Importantly, immune status plays a pivotal role in determining disease presentation and outcome. Immunocompetent patients often present with localized pulmonary disease and favorable outcomes, whereas immunocompromised patients—particularly those with severe immunodeficiency—are more likely to develop disseminated disease and experience higher mortality [2, 4]. Early and accurate diagnosis is essential for improving prognosis. The cryptococcal antigen (CrAg) assay has become the cornerstone of diagnosis, with both qualitative lateral flow tests and quantitative enzyme-linked immunosorbent assay (ELISA)-based methods demonstrating high sensitivity. However, discrepancies exist between the two methods, and their relative clinical value remains an area of active investigation [3, 5].

Despite advances in antifungal therapy, including the development of less nephrotoxic liposomal amphotericin B formulations, the use of single-dose liposomal amphotericin B combined with flucytosine and fluconazole, and improved azole-based treatment strategies, mortality remains unacceptably high in certain patient subgroups, particularly those with cryptococcal meningitis or disseminated disease [6–8]. Amphotericin B-based induction therapy is recommended for severe disease, but it is limited by toxicity and resource constraints, while azole monotherapy is widely used for pulmonary cryptococcosis [9]. A better understanding of the clinical characteristics, immune status distribution, diagnostic performance of CrAg assays, and prognostic factors in cryptococcosis is urgently needed, especially in non-HIV populations.

Therefore, we conducted an ambispective cohort study of 396 patients with cryptococcosis from 48 hospitals in China to clarify clinical characteristics, immune stratification, diagnostic performance, treatment strategies, and prognostic determinants.

## Methods

### Study design and participants

This was an ambispective cohort study including 396 HIV-negative patients with cryptococcosis diagnosed in 48 hospitals between 2013 and 2025. Patients from Fujian Province diagnosed between 2017 and 2022 were prospectively enrolled and followed, whereas patients from Jiangsu Province diagnosed between 2013 and 2025 were identified retrospectively through electronic medical records. The 48 hospitals were selected from Jiangsu and Fujian provinces in southeastern China because these regions have been consistently reported as endemic areas for cryptococcosis in China. Although not fully representative of all regions nationwide, these provinces reasonably reflect the current real-world situation of cryptococcosis management in China.

**Fig. 1:**
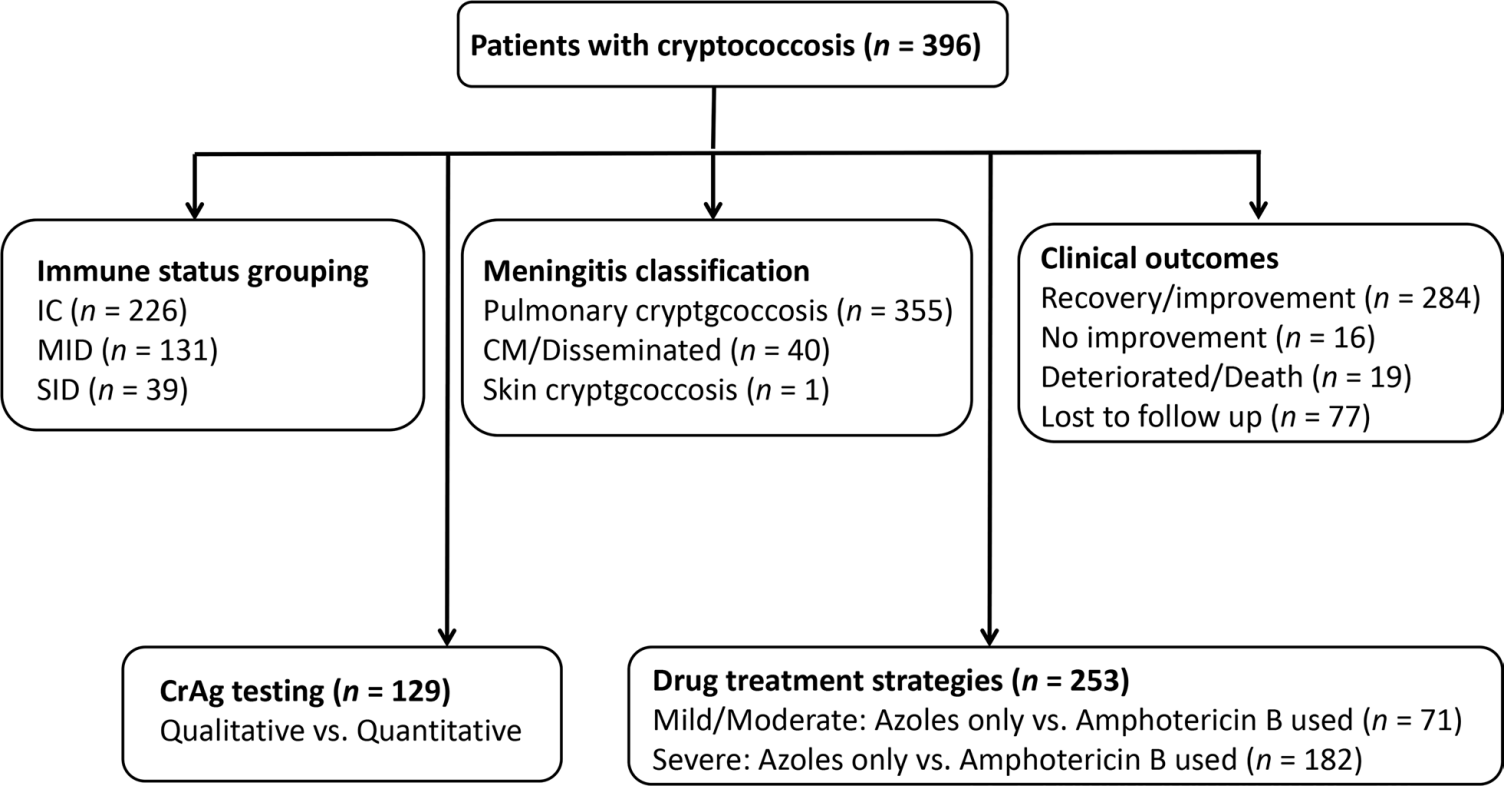
Study flowchart. The flow diagram illustrates the enrollment of 396 patients with cryptococcosis, stratification by immune status, disease classification, diagnostic evaluations, treatment regimens, and follow-up outcomes. *CM* Cryptococcal meningitis, *CrAg* Cryptococcal antigen, *IC* Immunocompetent, *MID* Mild-to-moderate immunodeficient, *SID* Severe immunodeficient

Eligibility was based on diagnostic criteria defined by the European Confederation of Medical Mycology (ECMM)/International Society for Human and Animal Mycology (ISHAM) global guidelines, incorporating clinical manifestations, radiological findings, microbiological evidence, and/or histopathological confirmation. All patients underwent routine HIV screening at the time of diagnosis, in accordance with standard clinical practice across participating centers. Exclusion criteria included incomplete clinical data or uncertain diagnosis. The study workflow, including patient enrollment, stratification by immune status and disease severity, diagnostic evaluation, and treatment allocation, is depicted in Fig. 1.

### Data collection

Demographic information (age, sex, smoking history), immune status, comorbidities, site of infection, and clinical symptoms at presentation were extracted from electronic medical records. Thoracic imaging (chest CT) was routinely performed for all patients at diagnosis to evaluate pulmonary involvement. Detailed radiologic characteristics of this cohort have been reported previously [10]. Patients were stratified by immune status [immunocompetent (IC), mild-to-moderate immunodeficient (MID), or severe immunodeficient (SID), according to comorbidities, immunosuppressive therapy, and laboratory findings], by disease type (pulmonary cryptococcosis, cryptococcal meningitis, or disseminated cryptococcosis), and by disease severity (mild-to-moderate vs. severe pulmonary cryptococcosis, as defined by ECMM/ISHAM guidelines). The severity of pulmonary cryptococcosis was classified as mild or severe according to clinical and radiological features, based on the principles of the 2024 ECMM/ISHAM/American Society for Microbiology (ASM) global guideline and our institutional criteria (Supplementary Appendix) [11]. SID was defined according to established clinical criteria, including: (1) CD4^+^ T-cell count < 200 cells/µl; (2) hematologic malignancies; (3) solid-organ or hematopoietic stem cell transplantation; (4) agranulocytosis (neutrophil count < 1.5 × 10^9^/L) due to radiotherapy, chemotherapy, or other causes; or (5) receipt of high-dose glucocorticoids (prolonged systemic corticosteroid therapy equivalent to ≥ 20 mg/day of prednisone for ≥ 3 weeks), strong immunosuppressive regimens, or other diseases and therapies associated with significant immune impairment.

### Laboratory and radiological examinations

Routine laboratory tests included complete blood counts and serum C-reactive protein (CRP) levels. Serum CrAg was tested in most patients, using a qualitative lateral flow assay (LFA) and, when available, a quantitative ELISA, with a positivity threshold of ≥ 5 µg/L. Lumbar puncture (LP) was performed in patients with suspected meningitis, and cryptococcal meningitis (CM) was confirmed by the identification of *Cryptococcus* in cerebrospinal fluid (CSF) using direct examination with China ink and by positive culture on Sabouraud’s medium. Pulmonary tissue biopsy (percutaneous lung biopsy or bronchoscopic biopsy) was performed in patients whose chest CT demonstrated lesions suspicious for pulmonary cryptococcosis, particularly when initial microbiologic tests (smear, culture, or serum CrAg) were inconclusive or when histopathologic confirmation was clinically indicated. Brain magnetic resonance imaging (MRI) was conducted in cases with neurological symptoms or suspected central nervous system involvement.

### Treatment, follow-up and prognosis

For immunocompetent patients with asymptomatic isolated pulmonary cryptococcosis, azole therapy (fluconazole 200–400 mg/day) was typically administered for 3–6 months. Symptomatic pulmonary disease was treated with fluconazole 400 mg/day for 6–12 months. A subset of patients with severe pulmonary involvement received amphotericin B-based induction regimens. The duration of therapy for patients with cryptococcal meningitis or disseminated cryptococcosis followed the same principles as above, generally requiring prolonged courses consistent with international guideline recommendations. In the retrospective cohort, some patients with meningitis or disseminated disease were documented as receiving azole monotherapy based on electronic medical records. As treatment information was retrospectively extracted, incomplete documentation cannot be excluded. To address this, treatment analyses were limited to patients with clearly documented antifungal regimens, and cases with missing or ambiguous treatment records were excluded from treatment-related analyses. When available, fluconazole was the primary agent used, typically at 400–800 mg/day. For the prospective arm of the study, patients were followed for up to 12 months. For the retrospective cohort, follow-up duration was determined by all available electronic medical records, which varied widely from several days to several years. Clinical outcomes were categorized into five grades based on symptoms, laboratory and imaging findings, and treatment response: recovery, improvement, no response/poor response, deteriorated, and deceased. Recovery was defined as complete resolution of symptoms and radiological abnormalities; improvement as partial resolution with mild residual findings; no response/poor response as little or no clinical or radiological improvement; deteriorated as worsening of disease; and deceased as all-cause mortality during follow-up.

### Statistical analysis

Continuous variables were summarized as median (interquartile range, IQR) and compared using the Mann-Whitney U test or Kruskal-Wallis test, as appropriate. Categorical variables were expressed as counts and percentages, and differences between groups were assessed by the Chi-square test or Fisher’s exact test. Concordance between qualitative (LFA) and quantitative (ELISA) serum CrAg assays was assessed using cross-tabulation and overall percent agreement. Statistical significance was set at *P* < 0.05. Analyses were performed using SPSS version 26.0 (IBM Corp., Armonk, NY, USA).

## Results

### Baseline characteristics

A total of 396 patients with cryptococcosis were included (Table 1). The median age was 52 years (IQR: 42–63), and 61.9% were male. With respect to immune status, 57.1% were immunocompetent, 33.1% had mild immunodeficiency, and 9.9% had severe immunodeficiency. Pulmonary disease predominated (89.7%), whereas 10.1% had meningitis or disseminated involvement. A wide range of comorbidities was observed, including diabetes mellitus, chronic kidney disease, chronic liver disease, autoimmune disorders, malignancy, etc., particularly in immunodeficient patients (Table 2).

**Table 1:** Baseline demographic, clinical, laboratory, and disease characteristics of non-HIV patients with cryptococcosis in a multi-center cohort study

Variable	Total (*n* = 396)
Age (years, median [IQR])	52.0 [42.0, 63.0]
Male	245 (61.9%)
Smoking status	
Yes	57 (14.4%)
No	332 (83.8%)
Unknown	7 (1.8%)
Immune status	
IC	226 (57.1%)
MID	131 (33.1%)
SID	39 (9.9%)
Site(s) of infection	
Lung only	355(89.7%)
Brain involvement	40(10.1%)
Skin	1(0.3%)
Comorbidities	262(66.2%)
Presenting signs/Symptoms	
No symptoms	130 (32.8%)
Fever ≥ 38 °C	60 (15.2%)
Respiratory symptoms (cough, sputum production, and/or dyspnea)	214 (54.0%)
Neurological symptoms	34 (8.6%)
Lymphocyte count (× 10^9^/L), median [IQR]	1.64 [1.2,2.1]
CRP level, mg/L, median [IQR]	6.77 [1.4,15.1]
Chest CT	181 (45.7%)
Nodules	131 (72.4%)
Patchy infiltrates	112 (61.9%)
LP	85 (21.5%)
Positive CSF smear	19 (22.4%)
Brain MRI examination	100 (25.3%)
Signs of meningitis	6 (6.0%)
Diagnostic workup	
Smear (CSF, BALF, et al.)	20/87 (23.0%)
Positive cultures (CSF, blood, BALF, lung tissues, et al.)	18/23 (78.3%)
mNGS	43/55 (78.2%)
Positive serum CrAg (qualitative)	245/266 (92.1%)
Positive serum CrAg (quantitative)	129/135 (95.6%)
Histopathology	152/174 (87.4%)
Drug treatment	***n* = 352**
Amphotericin B used	28 (8.0%)
Fluconazole or voriconazole used only	322 (91.5%)
Fluconazole + 5-fluorocytosine	2 (0.6%)
Prognosis	***n* = 319**
Recovered/Improved	284 (89.0%)
No improvement	16 (5.0%)
Deteriorated/Died	19 (6.0%)

*BALF* Bronchoalveolar lavage fluid, *CM* Cryptococcal meningitis, *CRP* C-reactive protein, *CrAg* Cryptococcal antigen, *CSF* Cerebrospinal fluid, *IC* Immunocompetent, *MID* Mild-to-moderate immunodeficient, *mNGS* Metagenomic next-generation sequencing, *SID* Severe immunodeficient

**Table 2:** Characteristics of 396 patients with cryptococcosis with different immune status

Variable	IC (*n* = 226)	MID (*n* = 131)	SID *(n* = *39)*	*P* value			
				Overall	IC vs. MID	IC vs. SID	MID vs. SID
Age (years, median [IQR])	47.0 [38.0, 57.0]	60.0 [52.0, 69.0]	52.0 [41.5, 62.0]	< 0.001	< 0.001	0.127	0.020
Male sex	147 (65.04%)	72 (54.96%)	26 (66.67%)	0.136	0.071	> 0.999	0.268
Smoking status							
Yes	29 (12.8%)	24 (18.3%)	4 (10.3%)	0.269	0.167	0.797	0.326
No	193 (85.4%)	104 (79.4%)	35 (89.7%)	0.190	0.146	0.620	0.164
Unknown	4 (1.8%)	3 (2.3%)	0 (0)	0.635	0.710	> 0.999	> 0.999
Site(s) of infection							
Lung only	212 (93.8%)	118 (90.1%)	25 (64.1%)	< 0.001	0.217	< 0.001	0.0003
Brain involvement	14 (6.2%)	13 (9.9%)	13 (33.3%)	< 0.001	0.217	< 0.001	0.002
Skin	0 (0)	0 (0)	1 (2.6%)	0.010	> 0.999	0.147	0.229
Comorbidities	94 (41.6%)	129 (98.5%)	39 (100%)	< 0.001	< 0.001	< 0.001	> 0.999
Presenting signs/Symptoms							
No symptoms	80 (35.4%)	44 (33.6%)	6 (15.4%)	0.048	0.818	0.015	0.029
Fever ≥ 38 °C	25 (11.1%)	23 (17.6%)	12 (30.8%)	0.004	0.107	0.004	0.112
Respiratory symptoms (cough, sputum production, and/or dyspnea)	126 (55.8%)	69 (52.7%)	19 (48.7%)	0.667	0.583	0.487	0.717
Neurological symptoms	13 (5.8%)	13 (9.9%)	8(20.5%)	0.008	0.204	0.005	0.096
Lymphocyte count (× 10^9^/L), median [IQR]	1.78 [1.4,2.2]	1.49 [1.0,2.0]	1.32 [0.8, 1.9]	< 0.001	< 0.001	0.004	0.487
CRP level, mg/L, median [IQR]	3.53 [0.8,10.0]	7.20 [2.3,17.4]	21.00 [7.2, 83.5]	< 0.001	0.006	< 0.001	0.009
LP	44 (19.5%)	28 (21.4%)	13 (33.3%)	0.150	0.683	0.059	0.139
Positive CSF smear	6 (13.6%)	9 (32.1%)	4 (30.8%)	0.0487	0.0968	0.0437	0.498
Brain MRI examination	4 (1.8%)	1 (0.8%)	1 (2.6%)	0.643	0.656	0.552	0.407
Signs of meningitis	1 (25.0%)	0 (0)	1 (100%)	0.137	> 0.999	0.273	0.229
Diagnostic workup							
Smear (CSF, BALF, et al.)	7/46 (15.2%)	9/28 (32.1%)	4/13 (30.8%)	0.188	0.144	0.237	> 0.999
Positive cultures (CSF, blood, BALF, lung tissues, et al.)	10/12 (83.3%)	3/6 (50.0%)	5/5 (100%)	0.112	0.268	> 0.999	0.182
mNGS	14/16 (87.5%)	22/31 (71.0%)	7/8 (87.5%)	0.338	0.287	> 0.999	0.653
Positive serum CrAg (qualitative)	146/155 (94.2%)	86/97 (88.7%)	13/14 (7.1%)	0.283	0.150	0.589	> 0.999
Positive serum CrAg (quantitative)	86/89(96.6%)	38/41(92.7%)	5/5 (100%)	0.530	0.379	> 0.999	> 0.999
Histopathology	95/103 (92.2%)	45/58 (77.6%)	12/13 (92.3%)	0.023	0.013	> 0.999	0.441
Drug treatment	*n* = 197	*n* = 121	*n* = 34				
Amphotericin B used	9 (4.6%)	13 (10.7%)	6 (17.7%)	0.011	0.038	0.013	0.386
Fluconazole or voriconazole used only	186 (94.4%)	108 (89.3%)	28 (82.4%)	0.275	> 0.999	0.129	0.172
Fluconazole + 5-fluorocytosine	2(1.0%)	0(0)	0(0)	0.470	0.534	> 0.999	> 0.999
Prognosis	*n* = 179	*n* = 106	*n* = 34				
Recovered/Improved	166 (92.7%)	93 (87.7%)	25 (73.5%)	0.476	0.624	0.248	0.433
No improvement	9 (5.0%)	5 (4.7%)	2 (5.9%)	0.933	> 0.999	0.668	0.661
Deteriorated/Died	4(2.2%)	8 (7.6%)	7 (20.6%)	< 0.001	0.036	< 0.001	0.049

*BALF* Bronchoalveolar lavage fluid, *CRP* C-reactive protein, *CrAg* Cryptococcal antigen, *CSF* Cerebrospinal fluid, *IC* Immunocompetent, *LP* Lumbar puncture, *MID* Mild-to-moderate immunodeficient, *mNGS* Metagenomic next-generation sequencing, *MRI* Magnetic resonance imaging, *SID* Severe immunodeficient

At presentation, 32.8% were asymptomatic. Respiratory symptoms were most common (54.0%), followed by fever ≥ 38 °C (15.2%) and neurological manifestations (8.6%). Median lymphocyte count was 1.64 × 10⁹/L (IQR: 1.19–2.10), and CRP was 6.77 mg/L (IQR: 1.37–15.08). Among patients who underwent thoracic imaging, chest CT most commonly demonstrated pulmonary nodules (131/181, 72.38%) and patchy infiltrates (112/181, 61.88%). LP was performed in 21.5% of patients, yielding a positive CSF smear in 22.4%, while brain MRI showed meningitis-related changes in 6.0%. Microbiological testing confirmed infection by smear (23.0%), culture (78.3%), and next-generation sequencing (78.2%). Serum CrAg assays showed high sensitivity, with 92.1% positive by qualitative and 95.6% by quantitative ELISA. Histopathological confirmation was obtained in 87.4% of biopsied specimens.

Among 352 patients with treatment records, azole monotherapy was the main regimen (91.5%), while 8.0% received amphotericin B-containing therapy and 0.6% fluconazole plus 5-fluorocytosine. Follow-up data were available in 319 patients: most improved or recovered, while a minority experienced no improvement or deterioration (Table 4).

### Clinical characteristics stratified by immune status

Significant differences were observed across immune status groups (Table 2). IC patients were younger (median 47 years) than MID (60 years) and SID (52 years, *P* < 0.001). Lung-limited disease predominated in IC and MID (> 90%) but was substantially lower in SID (64.1%, *P* < 0.001), whereas brain involvement was markedly higher in SID (33.3% vs. 6.2% IC and 9.9% MID, *P* < 0.001). Comorbidities were nearly universal among immunodeficient patients (MID 98.5%, SID 100%) compared with IC (41.6%, *P* < 0.001).

Clinical presentation also varied. Asymptomatic infection was more common in IC (35.4%) but less frequent in SID (15.4%, *P* = 0.048). Fever was enriched in SID (30.8%) versus IC (11.1%) and MID (17.6%, *P* = 0.004). Neurological symptoms were significantly more frequent in SID (20.5%) than IC (5.8%) or MID (9.9%, *P* = 0.008), while respiratory symptoms occurred at similar rates across groups (~ 50%). Lymphocyte counts were highest in IC and lowest in SID (*P* < 0.001), whereas CRP levels increased progressively with immune suppression (median 3.5, 7.2, and 21.0 mg/L, *P* < 0.001). Diagnostic yields showed similar trends across groups. CSF smears were more often positive in MID and SID (~ 30%) than IC (13.6%, *P* = 0.049). Serum CrAg positivity exceeded 88% in all groups, with quantitative assays detecting slightly more cases. Histopathology confirmed most diagnoses but was less frequent in MID (77.6%) than IC or SID (> 92%, *P* = 0.023).

Treatment patterns differed: amphotericin B-containing regimens were used more often in SID (17.7%) than MID (10.7%) or IC (4.6%, *P* = 0.011), although azole monotherapy remained predominant across all groups. Outcomes were best in IC (92.7% recovery/improvement) and MID (87.7%), but significantly worse in SID (73.5%), with higher rates of deterioration/death (20.6% vs. 2.2% IC and 7.6% MID, *P* < 0.001).

### Clinical characteristics according to presence of meningitis

Among 395 patients, 355 (89.9%) had pulmonary cryptococcosis, while 40 (10.1%) presented with meningitis or disseminated disease (Table 3). Patients with meningitis/dissemination were older and more likely to have severe immunodeficiency (32.5% vs. 7.0%) and comorbidities (97.5% vs. 62.5%).

**Table 3:** Characteristics of 395 patients with cryptococcosis according to presence or absence of meningitis

Variable	Pulmonary cryptococcosis (n = 355)	CM/Disseminated cryptococcosis(n = 40)	*P* value
Age (years, median [IQR])	52.0 [42.0, 62.0]	61.5 [44.0, 73.0]	0.053
Male	217 (61.1%)	28 (70.0%)	0.306
Smoking history			
Yes	51 (14.4%)	6 (15.0%)	0.817
No	297 (83.7%)	34 (85.0%)	> 0.999
Unknown	7 (2.0%)	0 (0.0%)	> 0.999
Immune status			
IC	212 (59.7%)	14 (35.0%)	0.004
MID	118 (33.2%)	13 (32.5%)	> 0.999
SID	25 (7.0%)	13 (32.5%)	< 0.001
Comorbidities	222(62.5%)	39 (97.5%)	< 0.001
Presenting signs/Symptoms			
No symptoms	123 (34.7%)	7 (17.5%)	0.033
Fever ≥ 38 °C	37 (10.4%)	23 (57.5%)	0.018
Respiratory symptoms (cough, sputum production, and/or dyspnea)	206 (58.0%)	8 (20.0%)	< 0.001
Neurological symptoms	6 (1.7%)	28 (70.0%)	< 0.001
Lymphocyte count (× 10^9^/L), median [IQR]	1.7 [1.3,2.1]	0.9 [0.6,1.4]	< 0.001
CRP level, mg/L, median [IQR]	6.1 [1.2,12.2]	25.1 [7.1,98.2]	0.002
LP	52 (14.7%)	33 (82.5%)	< 0.001
Positive CSF smear	0 (0)	19 (57.6%)	< 0.001
Brain MRI examination	84 (23.7%)	16 (40.0%)	0.034
Signs of meningitis	3 (3.6%)	3 (18.8%)	0.016
Diagnostic workup			
Smear (CSF, BALF, et al.)	0/53 (0)	20/34 (58.8%)	< 0.001
Positive cultures (CSF, blood, BALF, lung tissues, et al.)	15/19 (79.0%)	2/3 (66.7%)	0.686
mNGS	34/46 (73.9%)	9/9 (100%)	0.027
Positive serum CrAg (qualitative)	230/250 (92.0%)	15/16 (93.8%)	0.001
Positive serum CrAg (quantitative)	127/133 (95.5%)	2/2 (100%)	< 0.001
Histopathology	148/170 (87.1%)	3/3 (100%)	< 0.001
Drug treatment	***n* = 319**	***n* = 34**	
Amphotericin B used, n (%)	9 (2.8%)	19 (55.9%)	< 0.001
Fluconazole or voriconazole used only, n (%)	309 (96.9%)	13 (38.2%)	< 0.0001
Fluconazole + 5-fluorocytosine	0(0)	2 (5.9%)	0.010
Prognosis	***n* = 281**	***n* = 37**	
Recovered/Improved	263 (93.6%)	20 (54.1%)	0.003
No improvement	12 (4.3%)	4 (10.8%)	0.101
Deteriorated/Died	6 (2.1%)	13 (35.1%)	< 0.001

*BALF* Bronchoalveolar lavage fluid, *CRP* C-reactive protein, *CrAg* Cryptococcal antigen, *CSF* Cerebrospinal fluid, *IC* Immunocompetent, *LP* Lumbar puncture, *MID* Mild-to-moderate immunodeficient, *mNGS* Metagenomic next-generation sequencing, *MRI* Magnetic resonance imaging, *SID* Severe immunodeficient

Clinical features differed markedly. Asymptomatic infection was less frequent in meningitis/disseminated cases (17.5% vs. 34.7%), whereas fever (57.5% vs. 10.4%) and neurological symptoms (70.0% vs. 1.7%) were strongly enriched. Respiratory symptoms predominated in pulmonary disease (58.0%) but were uncommon in meningitis/dissemination (20.0%). Laboratory and imaging findings were also distinct. Meningitis/disseminated patients had lower lymphocyte counts, higher CRP, more frequent LP (82.5% vs. 14.7%), higher CSF smear positivity (57.6%), and more frequent meningitis-related MRI changes. Diagnostic yield was generally higher in meningitis/dissemination: smear positivity was markedly greater (58.8% vs. 0%), and NGS detected cryptococcal DNA in all tested cases. CrAg assays were highly sensitive in both groups (> 92%), with quantitative assays slightly superior.

Treatment patterns differed significantly. Amphotericin B-containing regimens were common in meningitis/disseminated disease (55.9%) but rare in pulmonary cases (2.8%), where azole monotherapy predominated (96.9%). Prognosis was substantially worse in meningitis/disseminated disease, with 35.1% mortality compared to only 2.1% in pulmonary cryptococcosis (*P* < 0.001).

### Prognostic differences

Of 319 patients with follow-up, 89.0% recovered or improved, 5.0% showed no improvement, and 6.0% deteriorated or died (Table 4). Poor prognosis was associated with older age, severe immunodeficiency (36.8% vs. 8.8% in improved), and CNS involvement (68.4% vs. 7.4%, all *P* < 0.001).

**Table 4:** Characteristics of 319 patients with cryptococcosis with different prognosis

Variable	Recovered/Improved (*n* = 284)	No improvement (*n* = 16)	Deteriorated/Died (*n* = 19)	*P* value			
				Overall	Recovered/Improved vs. No improvement	Recovered/Improved vs. Deteriorated/Died	No improvement vs. Deteriorated/Died
Age (years, median [IQR])	62.5 [52.0,74.3]	48.5 [35.8,58.3]	67.5 [59.5,73.3]	< 0.001	< 0.001	< 0.001	< 0.001
Male	177 (62.3%)	10 (62.5%)	13 (68.4%)	0.868	> 0.999	0.774	0.992
Smoking status							
Yes	41 (14.4%)	1 (6.3%)	5 (26.3%)	0.227	0.708	0.286	0.187
No	242 (85.2%)	15 (93.8%)	14 (73.7%)	0.238	0.484	0.309	0.187
Unknown	1 (0.4%)	0 (0)	0 (0)	0.940	> 0.999	> 0.999	> 0.999
Immune status							
IC	166 (58.5%)	9 (56.3%)	4 (21.1%)	0.006	> 0.999	0.002	0.043
MID	93 (32.8%)	5(31.3%)	8 (42.1%)	0.693	> 0.999	0.558	0.756
SID	25 (8.8%)	2 (12.5%)	7 (36.8%)	0.001	0.645	0.001	0.135
Site(s) of infection							
Lung only	263 (92.6%)	12 (75.0%)	6 (31.6%)	< 0.001	0.035	< 0.001	0.018
Brain involvement	21(7.4%)	4 (25.0%)	13 (68.4%)	< 0.001	0.035	< 0.001	0.018
Skin	1 (0.4%)	0 (0)	0 (0)	0.940	> 0.999	> 0.999	> 0.999
Presenting signs/Symptoms							
No symptoms	91 (32.0%)	2 (12.5%)	2 (10.5%)	0.042	0.162	0.069	> 0.999
Fever ≥ 38 °C	45 (15.9%)	3 (18.8%)	9 (47.4%)	0.002	0.728	0.002	0.152
Respiratory symptoms (cough, sputum production, and/or dyspnea)	160 (56.3%)	8 (50.0%)	7 (36.8%)	0.235	0.812	0.157	0.659
Neurological symptoms	22 (7.8%)	3 (18.8%)	9 (47.4%)	< 0.001	0.139	< 0.001	0.152
Lymphocyte count (× 10^9^/L), median [IQR]	1.63 [1.2,2.1]	1.37 [0.8,1.7]	0.95 [0.7,1.4]	0.010	0.179	0.005	0.437
CRP level, mg/L, median [IQR]	5.32 [1.3,12.6]	4.03 [1.3,12.8]	51.90 [15.4,106.5]	0.009	0.803	0.002	0.039
LP	63 (22.2%)	6 (37.5%)	10 (52.6%)	0.006	0.266	0.006	0.579
Positive CSF smear	9 (14.3%)	0 (0)	6 (60.0%)	0.001	> 0.999	0.004	0.034
Diagnostic workup							
Smear (CSF, BALF, et al.)	9 (3.2%)	0 (0)	6 (31.6%)	0.342	> 0.999	0.393	0.457
Positive cultures (CSF, blood, BALF, lung tissues, et al.)	13 (4.6%)	0 (0)	5 (26.3%)	< 0.001	> 0.999	0.001	0.049
mNGS	34 (12.0%)	3 (18.8%)	2 (10.5%)	0.704	0.429	> 0.999	0.642
Positive serum CrAg (qualitative)	185 (65.1%)	7 (43.8%)	5 (26.3%)	0.001	0.142	0.002	0.468
Positive serum CrAg (quantitative)	84 (29.6%)	4 (25.0%)	1 (5.3%)	0.071	0.786	0.019	0.156
Histopathology	113 (39.8%)	6 (37.5%)	2 (10.5%)	0.039	> 0.999	0.013	0.105
Drug Treatment	***n* = 263**	***n* = 13**	***n* = 18**				
Amphotericin B used	18 (6.8%)	4 (30.8%)	5 (27.8%)	< 0.001	0.014	0.007	> 0.999
Fluconazole or voriconazole used only	244 (92.8%)	8 (61.5%)	13 (72.2%)	< 0.001	0.001	0.010	0.811
Fluconazole + 5-fluorocytosine	1 (0.4%)	1 (7.7%)	0 (0)	0.007	0.092	> 0.999	0.419

*BALF* Bronchoalveolar lavage fluid, *CRP* C-reactive protein, *CrAg* Cryptococcal antigen, *CSF* Cerebrospinal fluid, *IC* Immunocompetent, *LP* Lumbar puncture, *MID* Mild-to-moderate immunodeficient, *mNGS* Metagenomic next-generation sequencing, *SID* Severe immunodeficient

Clinical presentation varied. Asymptomatic disease was more common in recovered patients but rare in poor-outcome groups, while fever and neurological symptoms were strongly enriched among those who deteriorated or died. Respiratory symptoms were frequent across all groups without significant differences. Laboratory markers also differed: patients who died had the lowest lymphocyte counts and the highest CRP levels (both *P* < 0.01). LP and CSF smear positivity were more common in poor-outcome patients, consistent with more severe CNS involvement. Diagnostic yields showed similar patterns: smear positivity was higher in poor-outcome cases, while CrAg positivity was lower compared with recovered patients. Histopathology positivity was also reduced in those who died.

Treatment patterns differed by outcome. Most recovered patients received azole monotherapy, whereas amphotericin B was more frequently used among patients with poor prognosis, reflecting clinical severity rather than therapeutic benefit.

### Diagnostic consistency of CrAg testing

Comparison between CrAg qualitative (LFA) and quantitative (ELISA, threshold 5 µg/L) assays demonstrated high agreement (Table 5). Among 129 patients tested, 119 were positive in both assays, while 3 were positive only by qualitative and 4 positives only by quantitative methods. Overall, the concordance rate exceeded 94%, but the quantitative method identified additional positive cases that were missed by qualitative testing, highlighting its diagnostic value.

**Table 5:** Agreement between CrAg qualitative and quantitative assays in serum

	Positive CrAg (ELISA)	Negative CrAg (ELISA)	All
Positive CrAg (LFA)	119	3	122
Negative CrAg (LFA)	4	3	7
All	123	6	129

*CrAg* Cryptococcal antigen, *ELISA* Enzyme-linked immunosorbent assay, *LFA* Lateral flow assay

### Treatment and outcomes by pulmonary cryptococcosis severity

Among patients with mild-to-moderate Pulmonary Cryptococcosis (*n* = 71), most received fluconazole or voriconazole monotherapy (70 patients), achieving a 97.1% recovery rate. Only one patient showed no response, and one deteriorated. For severe cases (*n* = 182), amphotericin B was administered in 8 patients, yielding 75% recovery but 25% non-response. By contrast, 174 severe cases treated with azoles alone had a higher response rate (98.9%) with lower rates of non-response (4.0%) and deterioration (2.9%). No significant differences were observed between regimens (*P* > 0.05), though amphotericin B was preferentially used in more severe clinical presentations (Table 6).

**Table 6:** Treatment and outcomes of 253 patients with pulmonary cryptococcosis by severity according to ECMM/ISHAM guideline

Outcome/Therapy	Amphotericin B used (*n* = 9)	Fluconazole or voriconazole used only (*n* = 244)	*P*
Mild-to-moderate	***n* = 1**	***n* = 70**	
Recovery/Improvement	1 (100%)	68 (97.1%)	> 0.999
No response	0 (0)	1 (1.4%)	> 0.999
Deteriorated/Died	0 (0)	1 (1.4%)	> 0.999
Severe	***n* = 8**	***n* = 174**	
Recovery/Improvement	6 (75%)	172 (98.9%)	0.107
No response	2 (25%)	7 (4.0%)	0.052
Deteriorated/Died	0 (0)	5 (2.9%)	> 0.999

*ECMM* European Confederation of Medical Mycology, *ISHAM* International Society for Human and Animal Mycology

## Discussion

In this large ambispective cohort of 396 patients with cryptococcosis, we identified several important determinants of clinical outcomes. Host immune status, CNS involvement, and systemic inflammatory markers consistently stratified prognosis, with severe immunodeficiency and meningitis/dissemination associated with markedly higher mortality. Serum cryptococcal antigen assays demonstrated high overall sensitivity and good concordance, while quantitative testing provided incremental diagnostic yield but failed to detect a few cases identified by qualitative assays. In pulmonary disease, azole monotherapy remained the predominant treatment and was highly effective across severity groups, whereas amphotericin B was rarely used and largely reserved for severe cases. Collectively, these findings highlight the heterogeneity of cryptococcosis in non-HIV populations and underscore the need for tailored diagnostic and therapeutic approaches based on host background and disease severity.

Our findings align with and expand upon previous observations in non-HIV cryptococcosis cohorts, which indicated that patients with severe immunodeficiency more frequently present with fever and neurological manifestations, exhibit lower lymphocyte counts and elevated CRP, and suffer poorer outcomes, underscoreing the critical role of host immunity. What’s more, CNS involvement remains a grave prognostic factor-our meningitis/disseminated group showed more severe neurological symptoms, higher CSF smear positivity, and much higher mortality rate (35.1% vs. 2.1% in pulmonary disease). A recent study on CM outcomes similarly reported worse prognosis in non-HIV compared to HIV patients, highlighting the clinical challenge in non-HIV-associated CM [12]. This resonates with findings by Ssebambulidde et al., who emphasized the high morbidity and mortality of CM in immunosuppressed non-HIV populations (~ 30–50%), including patients with autoantibodies or other immune dysfunctions [13]. In a landmark retrospective study of 302 patients, Brizendine et al. reported that non-HIV, non-transplant (NHNT) status was associated with higher mortality compared to HIV-positive and transplant recipients, with cryptococcemia and elevated intracranial pressure predicting poor outcomes [2]. Our data extend this by demonstrating that within a non-HIV cohort, severe immunodeficiency, rather than immune competence, is a key driver of adverse prognosis. Immunological mechanisms may further explain these observations. Panackal et al. found paradoxical, overly intense CSF T-cell activation in apparently immunocompetent individuals with cryptococcal meningitis, coupled with inflammatory damage and poor fungal clearance [4]. This suggests that both immunodeficiency and dysfunctionally excessive immune responses can contribute to poor outcomes—a duality mirrored in our cohort’s broad spectrum of immune status and corresponding mortality.

In routine practice, many clinicians avoid LP in asymptomatic patients, citing low perceived risk of CNS dissemination, patient reluctance, and limited procedural resources. In our cohort, data on LP among pulmonary cryptococcosis were 14.56%, suggesting that most such patients did not undergo the procedure. Evidence from Uganda indicates that LP offers little additional benefit in asymptomatic individuals with low serum CrAg titers (≤ 1∶80), as empiric treatment for meningitis in this group did not improve outcomes [14]. In contrast, elevated serum CrAg titers (> 1∶160) have been shown to strongly predict CNS involvement and the need for intensive antifungal therapy [15]. These findings support a risk-stratified approach: patients with preserved immunity, low CrAg titers, and no neurological symptoms may safely defer LP, whereas those with high titers or neurological signs warrant prompt evaluation. And in immunodeficient patients with a positive serum CrAg test, LP should be performed whenever neurological symptoms are present, to promptly evaluate for CNS involvement and guide timely management. In our cohort, the predominant causes of immunodeficiency included diabetes mellitus, chronic kidney disease, chronic liver disease, autoimmune disorders, malignancy, and chronic immunosuppressive therapy. Although such diagnostic algorithms are already well established for advanced HIV-associated cryptococcal meningitis, similar risk-based approaches should also be adapted and applied to non-HIV immunodeficient patients, in whom standardized decision pathways are less consistently implemented.

Regarding diagnostic methods, prior evaluations of the IMMY CrAg LFA in HIV-negative populations demonstrated pooled sensitivities of 96–99%, affirming its utility beyond HIV-infected cohorts [16]. Moreover, Tang et al. recently reported strong agreement (> 99%) between CrAg LFA and a novel chemiluminescence immunoassay (CLIA), with CLIA offering advantages in treatment monitoring [17]. Our report of 94.6% concordance between qualitative and quantitative CrAg assays, with incremental detection by quantitative ELISA, is consistent with these findings and underscores the practical benefit of quantitation for early diagnosis or monitoring. However, discrepancies were observed. In our cohort, three confirmed cases were positive by LFA but negative by ELISA (0, 0, and 3.2 µg/L), representing false negatives of the quantitative assay. This likely reflects very low antigen levels near the ELISA detection threshold or possible *C. gattii* infection, which is detected by LFA but not by the *C. neoformans*-specific ELISA kit [5]. Conversely, four confirmed cases were negative by LFA but positive by ELISA (5–24 µg/L), consistent with false negatives of the qualitative assay. This pattern is most plausibly explained by the prozone (high-dose hook) effect at elevated antigen concentrations, where excess antigen prevents line formation in LFA, whereas ELISA—with dilution steps—can still detect the antigen [18]. Similar discordance has been reported in prior evaluations of CrAg assays, where immune complex formation, species differences, and hook effects accounted for divergent results [5, 19, 20]. Collectively, these findings indicate that neither assay is infallible: ELISA may miss low-level or *C. gattii* infections, while LFA may underperform in high-titer cases. Their complementary use may therefore enhance diagnostic accuracy across the clinical spectrum of cryptococcosis. Although *C. gattii* infection could theoretically contribute to ELISA false-negative results, we were unable to perform strain genotyping due to limited culture-positive samples. This possibility, which we have discussed, warrants further investigation in future studies incorporating molecular identification of *Cryptococcus* species.

In our cohort, mNGS demonstrated high sensitivity for *Cryptococcus*, especially in meningitis/disseminated cases, and frequently identified infection when smear or culture were negative. These findings are consistent with recent reports showing that mNGS can provide rapid and accurate detection of *Cryptococcus* in cerebrospinal fluid and respiratory specimens, improving diagnostic yield in culture-negative patients [21–23]. Nevertheless, the method has limitations, including high cost, variable turnaround time, and occasional false negatives in low fungal burden samples. Overall, mNGS represents a useful complement to CrAg assays and conventional methods, particularly in diagnostically challenging cases.

Our treatment findings diverge from current international recommendations. Both the Infectious Diseases Society of America (IDSA) guidelines and the 2019 ECMM/ISHAM global guideline recommend amphotericin B-based induction therapy, for severe pulmonary and CNS cryptococcosis because of its fungicidal activity and proven survival benefit [9, 11]. In our cohort, amphotericin B was rarely used, even in severe pulmonary disease (4.4%, 8/182), and almost never in mild-to-moderate cases (1.4%, 1/71). Instead, azole monotherapy—most commonly fluconazole or voriconazole—remained the predominant regimen, administered to over 95% of patients across severity groups. Clinical response rates were high with azoles in both mild-to-moderate (97.1%) and severe pulmonary cryptococcosis (98.9%), although treatment failure and non-response occurred more often in severe cases (4.0% vs. 1.4%). This discrepancy between guideline recommendations and real-world practice likely reflects concerns regarding amphotericin B toxicity, cost, and feasibility. The higher failure rate observed with azoles in severe disease may relate to higher fungal burden, the fungistatic rather than fungicidal nature of azoles, limited drug penetration into necrotic or fibrotic lesions, occasional reduced susceptibility, and impaired host immunity. Together, these findings underscore the need for more intensive induction therapy in patients with severe pulmonary cryptococcosis, as recommended by international guidelines [9, 11], while recognizing the pragmatic reliance on azole therapy in non-HIV populations. Similar observations have been reported in non-HIV cryptococcal meningitis, where high antigen titers and heavy fungal burden were associated with increased mortality despite fluconazole therapy [24].

Our findings carry several implications for clinical practice. First, risk stratification based on immune status, serum CRP, lymphocyte counts, and CNS involvement may aid in early identification of patients at greatest risk for poor outcomes. A stratified diagnostic and management approach should consider the full spectrum of immunodeficiency—not only HIV infection, but also common non-HIV causes such as diabetes mellitus, chronic kidney and liver diseases, autoimmune disorders, malignancy, and chronic immunosuppressive therapy, which were the predominant contributors to immune dysfunction in our cohort. Second, while serum CrAg testing remains highly sensitive, combining qualitative and quantitative assays can reduce false negatives, and mNGS may serve as an important adjunct in diagnostically challenging cases. Third, LP remains indispensable in patients with neurological symptoms or high serum CrAg titers, but a risk-stratified approach may spare asymptomatic, low-titer patients from unnecessary invasive procedures. And for patients with cryptococcal meningitis, LP was performed when clinically indicated. However, opening pressure values and detailed intracranial pressure (ICP)-lowering interventions were not consistently documented across centers, particularly in the retrospective cohort; therefore, ICP measurements could not be systematically analyzed. Finally, the discrepancies in treatment between guideline recommendations and real-world practice highlight the urgent need for pragmatic strategies that consider efficacy, safety, and resource availability, particularly in non-HIV populations.

This study has several limitations. First, its ambispective design may have introduced information bias. Second, although multicenter, all participating hospitals were located in two southeastern provinces of China, where cryptococcosis incidence is relatively high; thus, regional differences and heterogeneity in medical resources may limit generalizability. Third, treatment strategies were not randomized and may have been influenced by physician preference, introducing potential selection bias in outcome comparisons. Despite these limitations, our findings provide one of the most comprehensive evaluations of cryptococcosis in non-HIV Chinese populations to date.

## Conclusions

This large ambispective cohort of non-HIV cryptococcosis highlights immune status, CNS involvement, and systemic inflammation as key determinants of outcome. Quantitative and qualitative CrAg assays, together with mNGS, complement each other in improving diagnostic accuracy, while LP remains essential for patients with neurological symptoms or high antigen titers. Although international guidelines recommend amphotericin B-based induction therapy for severe pulmonary and CNS disease, our data demonstrate that azole monotherapy remains the predominant regimen in practice and achieves favorable outcomes for pulmonary cryptococcosis. These findings underscore the need for pragmatic, risk-stratified diagnostic and therapeutic strategies that reconcile guideline recommendations with real-world feasibility. Future studies should validate risk models incorporating host immunity, inflammatory markers, and antigen burden, and explore optimized treatment algorithms tailored to non-HIV populations.

## Supplementary Information

Supplementary material 1.

Supplementary material 2

## Data Availability

The datasets used and analyzed during the current study are available from the corresponding author on reasonable request.

## Abbreviations

ASM: American Society for Microbiology
BALF: Bronchoalveolar lavage fluid
CLIA: Chemiluminescence immunoassay
CM: Cryptococcal meningitis
CNS: Central nervous system
CRP: C-reactive protein
CrAg: Cryptococcal antigen
CSF: Cerebrospinal fluid
ECMM: European Confederation of Medical Mycology
ELISA: Enzyme-linked immunosorbent assay
IC: Immunocompetent
ICP: Intracranial pressure
IDSA: Infectious Diseases Society of America
IQR: Interquartile range
ISHAM: International Society for Human and Animal Mycology
LFA: Lateral flow assay
LP: Lumbar puncture
MID: Mild-to-moderate immunodeficient
mNGS: Metagenomic next-generation sequencing
MRI: Magnetic resonance imaging
NHNT: Non-HIV, non-transplant
SID: Severe immunodeficient
