# Supplementary material for: Clinical spectrum, immune status, and prognostic factors of cryptococcosis: insights from a large, multi-center, ambispective cohort study in southeastern China
Source: Infect Dis Poverty. 2026 Jan 4;15:1. doi: 10.1186/s40249-025-01408-3 (PMC12765305; doi:10.1186/s40249-025-01408-3)
Supplement: Supplementary file 2 — Supplementary material 2 [file 40249_2025_1408_MOESM2_ESM.docx]

**Appendix. Criteria for classifying the severity of isolated pulmonary cryptococcosis.**

Pulmonary cryptococcosis severity was classified according to clinical and radiological features, following ECMM/ISHAM guideline principles^1^.

**Mild isolated pulmonary cryptococcosis** was defined as asymptomatic or mildly symptomatic disease with a single small pulmonary nodule <2 cm in diameter and no hypoxemia, without lobar consolidation, cavitation, or multilobar involvement.

**Severe isolated pulmonary cryptococcosis** was defined by one or more of the following: multiple pulmonary lesions, large lesions ≥2 cm, lobar consolidation, cavitation, multilobar involvement, or the presence of hypoxemia, suggesting a high fungal burden or more extensive parenchymal damage.

**References**

1. Chang CC, Harrison TS, Bicanic TA, et al. Global guideline for the diagnosis and management of cryptococcosis: an initiative of the ECMM and ISHAM in cooperation with the ASM. *Lancet Infect Dis* 2024; **24**(8): e495-e512.
